# (*E*)-5-Styryl-1*H*-indole and (*E*)-6-Styrylquinoline Derivatives Serve as Probes for β-Amyloid Plaques

**DOI:** 10.3390/molecules17044252

**Published:** 2012-04-10

**Authors:** Yang Yang, Hong-Mei Jia, Bo-Li Liu

**Affiliations:** Key Laboratory of Radiopharmaceuticals, Ministry of Education, College of Chemistry, Beijing Normal University, Beijing 100875, China; Email: girlamy@mail.bnu.edu.cn

**Keywords:** Alzheimer’s disease, β-amyloid plaques, binding affinity, imaging agent, SPECT

## Abstract

We report the synthesis and biological evaluation of novel (*E*)-5-styryl-1*H*-indole and (*E*)-6-styrylquinoline derivatives as probes for imaging β-amyloid (Aβ) plaques. These derivatives showed binding affinities for Aβ_1–40_ aggregates with *K*_i_ values varying from 4.1 to 288.4 nM. (*E*)-5-(4-iodostyryl)-1*H*-indole (**8**) clearly stained Aβ plaques in the brain sections of Alzheimer’s disease (AD) model mice (APP/PS1). Furthermore, autoradiography for [^125^I]**8** displayed intense and specific labeling of Aβ plaques in the brain sections mentioned above with low background. In biodistribution experiments using normal mice [^125^I]**8** showed high initial brain uptake followed by rapid washout (4.27 and 0.64% ID/g at 2 and 30 min post injection, respectively). These findings suggests that [^123^I]**8** may be a potential SPECT imaging agent for detecting Aβ plaques in AD brain.

## 1. Introduction

Alzheimer’s disease (AD) is a kind of irreversible, progressive brain disease characterized by dementia, cognitive impairment and memory loss. Although currently the pathogenesis of AD is not completely understood, it is generally accepted that β-amyloid (Aβ) plaques is considered to be one of the biomarkers for early diagnosis of AD [1,2,3]. Therefore, *in vivo* imaging agent for Aβ plaques applicable for PET (positron emission tomography) or SPECT (single photon emission computed tomography) would be very useful for early diagnosis of AD and provide significant information to evaluate the efficacy of AD therapies [4,5].

**Figure 1:**
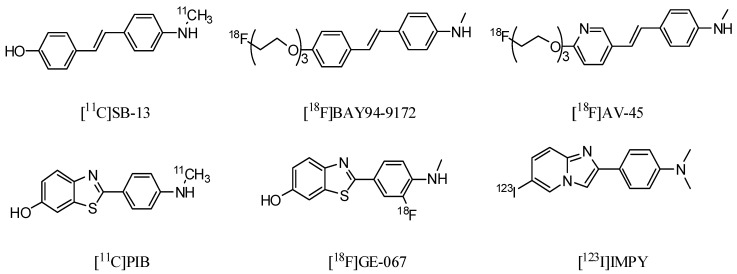
Chemical structures of Aβ imaging probes for clinical study.

To date, several radiolabeled ligands have been developed as imaging probes for Aβ plaques [6]. For example, [^11^C]SB-13 [7,8], [^18^F]BAY94-9172 [9] and [^18^F]AV-45 [10,11] derived from Congo Red (CR), [^11^C]PIB [12,13], [^18^F]GE-067 [14] and [^123^I]IMPY [15,16,17] derived from thioflavin T (ThT) (Figure 1). However, [^123^I]IMPY, the only SPECT tracer tested in human studies, has failed because of its low *in vivo* stability and its insufficient target-to-background ratio. In comparison with PET, SPECT is a more widely accessible and cost-effective technique in terms of routine diagnostic use. Consequently, the development of more useful imaging agents for Aβ plaques labeled with ^123^I (T_1/2_, 13 h, 159 keV) or ^99m^Tc (T_1/2_, 6 h, 140 keV) for SPECT has been a critical issue.

**Figure 2:**
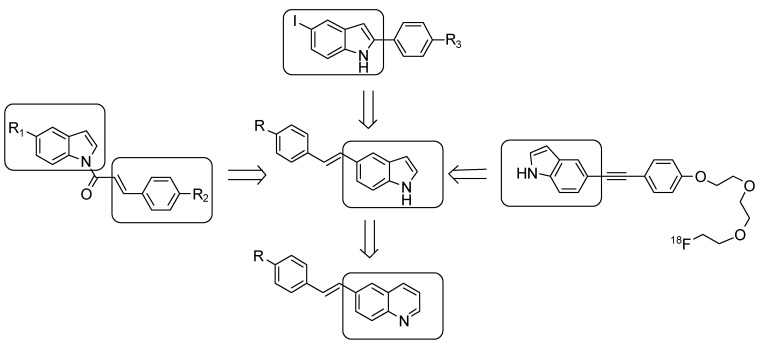
Design considerations of (*E*)-5-styryl-1*H*-indole and (*E*)-6-styrylquinoline derivatives.

Previously, we successfully developed a series of novel imaging agents for β-amyloid plaques based on the *N*-benzoylindole core which showed high binding affinities with *K*_i_ values in the nM range [18]. The brain uptake of these derivatives was encouraging, but their washout from the brain in normal mice appeared to be relatively slow. Qu *et al*. have developed indolylphenylacetylenes as potential Aβ plaques imaging agent, and the use of indolyl groups may improve the brain kinetics for β-amyloid imaging agents [19]. Recently, Watanabe *et al*. have developed phenylindoles for image β-amyloid in brain, these derivatives demonstrated high binding affinities to Aβ_1**–**4__2_aggregates [20]. Following these successful results, we applied highly conjugated (*E*)-5-styryl-1*H*-indole as a core structure for Aβ imaging agents to explore more useful candidates with favorable pharmacokinetics as Aβ imaging probes, and developed (*E*)-6-styrylquinoline derivatives for further studies (Figure 2). Reported herein are the synthesis and biological evaluation of novel (*E*)-5-styryl-1*H*-indole and (*E*)-6-styrylquinoline derivatives and especially, two radioiodinated derivatives as potential SPECT tracers for imaging β-amyloid plaques in the brain.

## 2. Results and Discussion

### 2.1. Chemistry and Radiochemistry

**Scheme 1:**
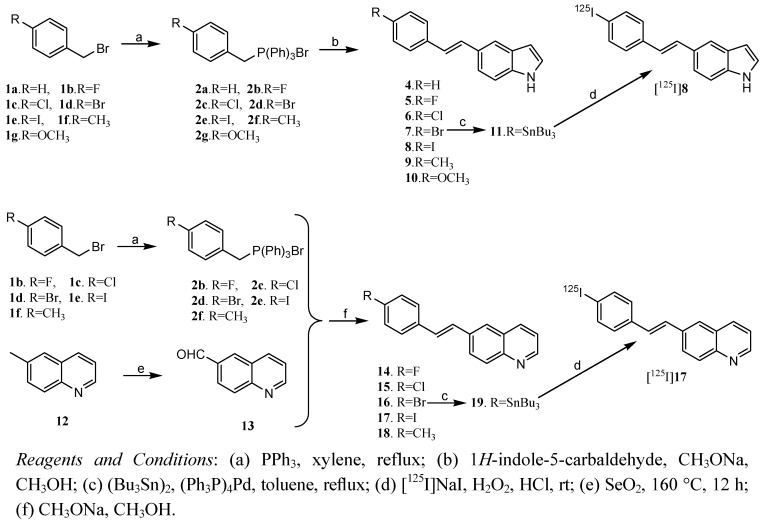
Synthetic route of (*E*)-5-styryl-1*H*-indole and (*E*)-6-Styrylquinoline derivatives.

The synthetic route to the (*E*)-5-styryl-1*H*-indole and (*E*)-6-styrylquinoline derivatives is shown in Scheme 1.

**Figure 3:**
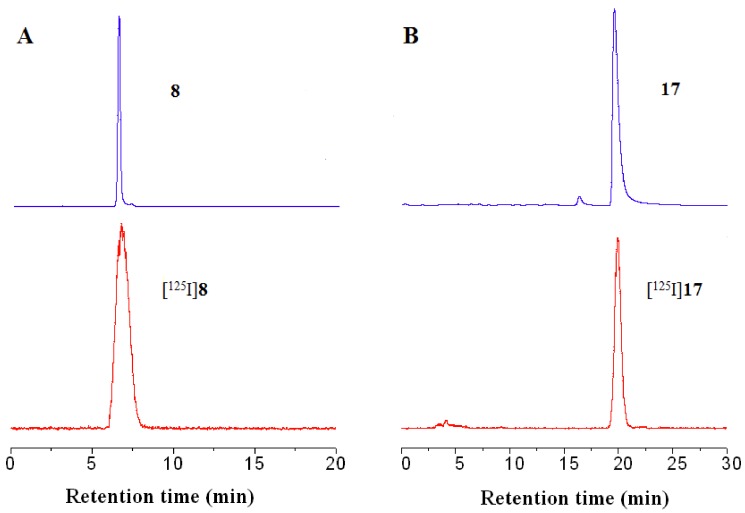
HPLC profiles of **8** (A, top), [^125^I]**8** (A, bottom) and **17** (B, top), [^125^I]**17** (B, bottom).

The key step was the base-catalyzed Wittig reaction between substituted triphenyl phosphonium ylides **2a**–**g** and 1*H*-indole-5-carbaldehyde or quinoline-6-carbaldehyde. The tributyltin derivatives **11**, **19** were prepared in yields of 22.3% and 28.6%, respectively, from the bromo-precursors **7**, **16**using an exchange reaction catalyzed by Pd(0). [^125^I]**8** and [^125^I]**17** were prepared via a iodo-destannylation reaction using hydrogen peroxide as the oxidant. The products were purified by radio-HPLC using a reverse-phase column and mobile phase consisting of acetonitrile with a flow rate of 1 mL/min. In order to identify the radiotracer, the non-radioactive **8** and **17** were co-injected and co-eluted with the corresponding radioactive product, respectively. Their HPLC profiles using acetonitrile and water (90:10 v/v) as mobile phase at a flow rate of 1 mL/min are present in Figure 3.

From Figure 3, the retention times of non-radioactive **8** and [^125^I]**8** were observed to be 6.45 min and 6.89 min, respectively. The retention times of non-radioactive **17** and [^125^I]**17** were observed to be 19.56 min and 19.94 min, respectively. The differences in retention time were in good agreement with the time lag which corresponds with the volume and flow rate within the distance between the UV and radioactive detector of our HPLC system. After purification by HPLC, the radiochemical purities of both [^125^I]**8** and [^125^I]**17** were greater than 98%. The radiochemical yields of [^125^I]**8** and [^125^I]**17** were 48–67% and 61–78%, respectively. The log *D* values of [^125^I]**8** and [^125^I]**17** were 2.52 ± 0.04 and 2.73 ± 0.03, respectively, which are in the appropriate range for brain imaging agents indicative of good permeability through the blood-brain barrier (BBB).

### 2.2. *In Vitro* Binding Studies Using the Aggregated Aβ_1–40_

The affinity of (*E*)-5-styryl-1*H*-indole and (*E*)-6-styrylquinoline derivatives for Aβ_1–40_ aggregates was determined by competition binding assay using [^125^I]TZDM as radio-ligand. TZDM was also screened using the same competition assay for comparison. The *K*_i_ values shown in Table 1 were varied from 4.1 to 288.4 nM suggesting that all these compounds share the same binding site with ThT. The *K*_i_ value of TZDM was 4.2 nM, which is comparable to that of previously reported in the literature (*K*_i_ = 0.9 nM) [21]. (*E*)-5-styryl-1*H*-indole (**4**) without any substituents showed moderate binding affinity (*K*_i_ = 25.1 nM). Introducing a F, Cl or OCH_3_ group at the *para*-position of the phenyl ring decreased the binding affinity (*K*_i_ = 89.3, 51.5 and 32.4 nM for compounds **5**, **6** and **10**, respectively), while introducing a Br or CH_3_ group at the same position increased the affinity (*K*_i_ = 16.3 and 15.8 nM for compounds **7** and **9**, respectively). It is noteworthy that compound **8** with a iodo group showed *K*_i_ value of 4.1 nM, which is comparable with that of TZDM. In general, (*E*)-5-styryl-1*H*-indole derivatives showed slightly better potency in binding to Aβ_1**–**40_ aggregates than (*E*)-6-styrylquinoline derivatives. Since derivatives **8** and **17** with iodine at the *para*-position of the phenyl ring displayed nanomolar affinities for Aβ_1–40_ aggregates, we prepared [^125^I]**8** and [^125^I]**17** for further evaluation as potential ligands for ^123^I-labeled SPECT imaging agents.

**Table 1:** *K*_i_ values of (*E*)-5-styryl-1*H*-indole and (*E*)-6-styrylquinoline derivatives for Aβ_1–40_ aggregates against [^125^I]TZDM.

Compound	*K*_i_ (nM) ^a^	Compound	*K*_i_ (nM) ^a^
**4**	25.1 ± 2.1	**10**	32.4 ± 1.9
**5**	89.3 ± 2.6	**14**	270.4 ± 1.5
**6**	51.5 ± 1.0	**15**	45.0 ± 1.3
**7**	16.3 ± 1.7	**16**	23.5 ± 1.3
**8**	4.1 ± 0.2	**17**	8.6 ± 1.2
**9**	15.8 ± 1.5	**18**	288.4 ± 1.3
**TZDM**	4.2 ± 0.4	**TZDM ^b^**	0.9 ± 0.2

^a^ Measured in triplicate with results given as the mean ± SD; ^b^ Data from [21].

### 2.3. *In Vitro* Fluorescent Staining of Amyloid Plaques in Brain Sections from Transgenic Mouse

**Figure 4:**
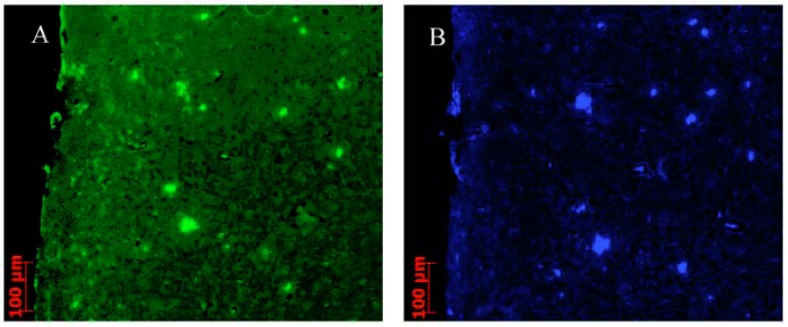
The labeled plaques were confirmed by staining of the adjacent sections by thioflavin-S (**A**); Fluorescence staining of compound **8** on AD model mouse sections from the cortex (**B**).

To confirm the binding affinities of these derivatives for Aβ plaques in the brain, *in vitro* fluorescent staining of brain sections (8 µm) from a transgenic model mouse (APP/PS1, 12 months, male) was carried out with compound **8**. As shown in Figure 4, many fluorescence spots were observed in the brain sections of transgenic mice (Figure 4B). The fluorescent labeling pattern was consistent with that observed with thioflavin-S (Figure 4A). These results suggested that **8** show specific binding to Aβplaques in the transgenic model mouse brain.

### 2.4. *In Vitro* Labeling of Brain Sections from Transgenic Mouse by Autoradiography

**Figure 5:**
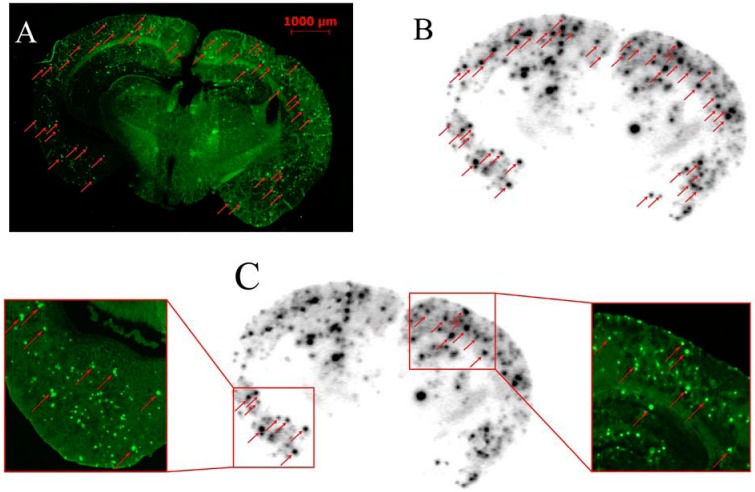
The presence and distribution of plaques in the sections were confirmed with thioflavin-S staining (**A**, **C**) (red arrows); Autoradiography of [^125^I]**8** *in* *vitro* in Tg model mouse (APP/PS1, 12 months, male) brain sections (**B**).

The results of *in vitro* autoradiography of [^125^I]**8** in the brain sections of a transgenic model mouse (APP/PS1, 12 months, male) are shown in Figure 5. [^125^I]**8** showed excellent labeling of Aβ plaques in the cortex region of the brain sections, and no remarkable accumulation of radioactivity were observed in white matter. The same sections were also stained with thioflavin-S and the localizations of Aβ plaques were in accord with the results of autoradiography. These results demonstrated that [^125^I]**8** was specific for Aβ plaques, which were consistent with the high binding affinity of compound **8** to Aβ_1–40_ aggregates.

### 2.5. *In Vivo* Biodistribution Studies

*In vivo *biodistribution studies of [^125^I]**8** and [^125^I]**17** were performed in normal mice. The uptake of radiotracer in the organs of interest at different time points after intravenous administration of [^125^I]**8** and [^125^I]**17** is summarized in Table 2. [^125^I]**8** showed high initial brain uptake followed by rapid clearance (4.27 and 0.28% ID/g at 2 and 60 min post injection, respectively). On the other hand, [^125^I]**17** showed relatively low brain uptake and slow washout (2.05 and 0.55% ID/g at 2 and 60 min post injection, respectively). As compared with previously reported radioiodinated *N*-benzoylindole derivatives [18], radioiodinated (*E*)-5-styryl-1*H*-indole derivative [^125^I]**8** showed greatly improved brain uptake. Because there are no plaques in normal brain, potential Aβ-specific probe should possess high brain uptake followed by fast washout in normal mice. The brain_2min_/brain_60min_ ratio has been used as an index to compare the washout rate from normal brain and select candidate tracers with appropriate kinetics *in vivo*. It was reported that [^123^I]IMPY showed a high initial brain uptake and fast washout in normal mice (2.88% ID/organ and 0.21% ID/organ at 2 min and 60 min postinjection, respectively) [16]. The brain_2min_/brain_60min_ ratio of [^125^I]**8** (15.3) is higher than that of [^123^I]IMPY (13.7), indicating [^125^I]**8** may possess suitable pharmacokinetic properties for imaging Aβ plaques in AD brain. Accordingly, [^125^I]**8** may be comparable or even better for detecting Aβ plaques. Therefore, (*E*)-5-styryl-1*H*-indole derivative [^125^I]**8**, with nanomolar affinity to Aβ_1**–**40_ aggregates, excellent BBB permeability as well as fast washout from the normal brain, may be suitable for development as a novel Aβ imaging agent.

**Table 2:** Biodistribution in normal mice after iv injection of [^125^I]**8** and [^125^I]**17** (% ID/g, avg of 5 mice ± SD) and its partition coefficient (D).

Organ	2 min	15 min	30 min	60 min	120 min	240 min
[^125^I]**8** (log *D* = 2.52 ± 0.04)						
Blood	11.91 ± 0.62	12.29 ± 1.30	7.64 ± 0.62	4.49 ± 0.28	2.93 ± 0.30	1.50 ± 0.13
Brain	4.27 ± 0.49	1.37 ± 0.16	0.64 ± 0.11	0.28 ± 0.06	0.20 ± 0.08	0.10 ± 0.02
Heart	5.76 ± 0.38	3.70 ± 0.40	2.55 ± 0.52	1.59 ± 0.28	1.47 ± 0.48	0.65 ± 0.16
Liver	14.73 ± 0.66	10.66 ± 0.31	7.14 ± 1.13	4.45 ± 0.23	4.19 ± 0.61	3.06 ± 0.36
Spleen	4.38 ± 0.33	4.21 ± 0.26	3.44 ± 0.21	2.44 ± 0.12	1.87 ± 0.28	1.37 ± 0.09
Lung	10.76 ± 0.63	8.22 ± 0.88	5.39 ± 0.83	3.28 ± 0.10	2.30 ± 0.20	1.43 ± 0.52
Kidney	11.66 ± 1.52	14.89 ± 4.23	9.46 ± 1.95	4.65 ± 0.98	1.93 ± 0.76	1.34 ± 0.36
Stomach ^a^	1.23 ± 0.56	4.82 ± 0.46	3.50 ± 0.19	1.72 ± 0.21	3.48 ± 0.79	2.31 ± 1.44
Muscle	2.64 ± 0.40	2.01 ± 0.24	1.25 ± 0.04	0.81 ± 0.12	0.61 ±0.24	0.35 ± 0.09
[^125^I]**17** (log *D* = 2.73 ± 0.03)						
Blood	11.39 ± 1.56	6.31 ± 0.51	5.80 ± 0.37	3.86 ± 0.74	1.97 ± 0.35	1.38 ± 0.24
Brain	2.05 ± 0.25	1.18 ± 0.17	0.93 ± 0.13	0.55 ± 0.11	0.26 ± 0.03	0.14 ± 0.02
Heart	7.70 ± 0.86	3.80 ± 0.12	3.21 ± 0.11	2.55 ± 0.21	1.33 ± 0.06	0.80 ± 0.19
Liver	22.45 ± 1.79	9.95 ± 0.18	9.12 ± 0.53	6.97 ± 0.28	3.93 ± 0.46	2.96 ± 0.28
Spleen	5.88 ± 0.30	5.91 ± 0.58	4.66 ± 0.52	4.71 ± 0.97	2.25 ± 0.36	1.59 ± 0.17
Lung	13.56 ± 1.71	6.42 ± 0.47	5.64 ± 0.41	4.55 ± 0.49	2.01 ± 0.18	1.43 ± 0.28
Kidney	15.01 ± 1.56	7.47 ± 1.01	6.57 ± 0.50	4.72 ± 0.69	2.20 ± 0.34	1.64 ± 0.23
Stomach ^a^	4.05 ± 0.09	15.84 ± 0.78	8.11 ± 1.21	6.75 ± 0.43	11.12 ± 2.48	7.03 ± 1.51
Muscle	2.78 ± 0.42	1.68 ± 0.20	2.30 ± 0.32	1.40 ± 0.34	0.72 ± 0.21	0.61 ± 0.08

^a^ Expressed as % ID/organ.

## 3. Experimental

### 3.1. General

Unless otherwise indicated, all chemicals used in synthesis were commercial products and were used without further puriﬁcation. Na^125^I (2200 Ci/mmol) was obtained from PerkinElmer Life and Analytical Sciences, USA. The double transgenic (APP/PS1) AD model mouse was obtained from Institute of Laboratory Animal Science, Chinese Academy of Medical Sciences and Comparative Medicine Center of Peking Union Medical College (Beijing, China). ^1^H-NMR spectra were obtained on Bruker (400 MHz) NMR spectrometer at room temperature with TMS as an internal standard. Chemical shifts are reported as δ values relative to internal TMS. Coupling constants are reported in hertz. The multiplicity is deﬁned by s (singlet), d (doublet), t (triplet), and m (multiplet). Mass spectra were acquired using the Surveyor MSQ Plus (ESI) (Waltham, MA, USA) instrument. HPLC was performed on a Shimadzu SCL-10AVP system (Shimadzu Corporation, Kyoto, Japan) which consisted of a binary pump with on-line degasser, a model SPD-10AVP UV-VIS detector operating at a wavelength of 254 nm, and a Packard 500TR series flow scintillation analyzer (Packard BioScience Co., Wallingford, CT, USA) with a Alltech Alltima RPC-18 column (5 μm, ID = 4.6 mm, length = 250 mm). The samples were analyzed using acetonitrile and water (90:10 v/v) as mobile phase at a flow rate of 1 mL/min. The sample was separated using acetonitrile as mobile phase at a flow rate of 1 mL/min. All key compounds were proven by analytical HPLC analysis to show ≥95% purity (Supporting information).

#### 3.1.1. General Procedure for Preparing Substituted Triphenyl Phosphonium Ylide **2** (**2a**–**g**)

The suitable 4-substituted-1-(bromomethyl)benzene **1a**–**g** (1 mmol) and triphenylphosphine (1 mmol) was heated to reflux in xylene (10 mL) for about 6 h. The mixture was filtered and crude materials were purified by recrystallization with toluene.

#### 3.1.2. General Procedure for Preparing **4**–**11**, **13**

The appropriate substituted compounds **2a**–**g** (1 mmol), 1*H*-indole-5-carbaldehyde (**3**, 1 mmol), and CH_3_ONa (1 mmol) was heated to reflux in CH_3_OH (12 mL) for about 5 h. The organic solvent was removed under vacuum. Crude materials were purified by column chromatography on silica gel (petroleum ether/AcOEt, 4/1).

*(E)-5-Styryl-1H-indole *(**4**). Yield 53.6%, ^1^H-NMR (DMSO-d_6_) δ: 7.73 (1H, s), 7.58 (2H, d, *J *= 7.5 Hz), 7.43–7.38 (5H, m), 7.32 (1H, d, *J* = 16.5 Hz), 7.22 (1H, t, *J* = 7.2 Hz), 7.12 (1H, d, *J* = 16.4 Hz), 6.44 (1H, d, *J *= 3.0 Hz). HRMS *m/z* C_16_H_13_N found 220.1120/calcd 220.1126 ([M+H]^+^). m.p. 162–163 °C.

*(E)-5-(4-Fluorostyryl)-1H-indole *(**5**). Yield 71.4%, ^1^H-NMR (DMSO-d_6_) δ: 7.72 (1H, s), 7.62 (2H, dd, *J_1_* = 8.6 Hz, *J_2_* =5.7 Hz), 7.40 (2H, dd, *J_1_* = 11.4 Hz, *J_2_* = 8.6 Hz), 7.34 (1H, d, *J* = 3.0 Hz), 7.27 (1H, d, *J* = 16.4 Hz), 7.19 (2H, t, *J *= 8.8 Hz), 7.12 (1H, d, *J *= 16.4 Hz), 6.44 (1H, d, *J *= 3.0 Hz). HRMS *m/z* C_16_H_12_FN found 238.0878/calcd 238.0876 ([M+H]^+^). m.p. 179–180 °C.

*(E)-5-(4-Chlorostyryl)-1H-indole *(**6**). Yield 70.2%, ^1^H-NMR (DMSO-d_6_) δ: 7.74 (1H, s), 7.60 (2H, d, *J *= 8.5 Hz), 7.44–7.38 (4H, m), 7.35 (1H, d, *J* = 3.0 Hz), 7.34 (1H, d, *J* = 16.4 Hz), 7.12 (1H, d, *J *= 16.4 Hz), 6.44 (1H, d, *J *= 3.0 Hz). HRMS *m/z* C_16_H_12_ClN found 254.0733/calcd 254.0737 ([M+H]^+^). m.p. 205–206 °C.

*(E)-5-(4-Bromostyryl)-1H-indole *(**7**). Yield 68.3%, ^1^H-NMR (DMSO-d_6_) δ: 7.74 (1H, s), 7.54 (4H, s), 7.43–7.40 (2H, m), 7.36 (1H, d, *J* = 16.4 Hz), 7.35 (1H, d, *J* = 3.1 Hz), 7.11 (1H, d, *J *= 16.4 Hz), 6.44 (1H, d, *J *= 3.0 Hz). HRMS *m/z* C_16_H_12_BrN found 298.0235/calcd 298.0231 ([M+H]^+^). m.p. 215–216 °C.

*(E)-5-(4-Iodostyryl)-1H-indole *(**8**). Yield 55.3%, ^1^H-NMR (DMSO-d_6_) δ: 7.73 (1H, s), 7.70 (2H, d, *J* = 8.4 Hz), 7.43–7.39 (4H, m), 7.36 (1H, d, *J* = 16.4 Hz), 7.34 (1H, d, *J* = 3.1 Hz), 7.08 (1H, d, *J *= 16.5 Hz), 6.44 (1H, d, *J *= 2.9 Hz). HRMS *m/z* C_16_H_12_IN found 246.1283/calcd 246.1299 ([M+H]^+^). m.p. 211–212 °C.

*(E)-5-(4-Methoxystyryl)-1H-indole *(**9**). Yield 67.7%, ^1^H-NMR (DMSO-d_6_) δ: 7.70 (1H, s), 7.47 (2H, d, *J *= 8.0 Hz), 7.39 (2H, dd, *J_1_* = 11.5 Hz, *J_2_* = 8.6 Hz), 7.33 (1H, d, *J* = 3.0 Hz), 7.25 (1H, d, *J* = 16.4 Hz), 7.17 (2H, d, *J *= 8.0 Hz), 7.08 (1H, d, *J *= 16.4 Hz), 6.44 (1H, d, *J *= 3.0 Hz), 2.31 (3H, s). HRMS *m/z* C_17_H_15_N found 234.1126/calcd 234.1126 ([M+H]^+^). m.p. 176–177 °C.

*(E)-5-(4-Methoxystyryl)-1H-indole *(**10**). Yield 65.2%, ^1^H-NMR (DMSO-d_6_) δ: 7.68 (1H, s), 7.51 (2H, d, *J *= 8.6 Hz), 7.40–7.35 (2H, m), 7.33 (1H, d, *J* = 3.0 Hz), 7.16 (1H, d, *J* = 16.4 Hz), 7.06 (1H, d, *J* = 16.4 Hz), 6.93 (2H, d, *J *= 8.5 Hz), 6.42 (1H, d, *J *= 3.0 Hz), 3.77 (3H, s). HRMS *m/z* C_17_H_15_NO found 250.1241/calcd 250.1232 ([M+H]^+^). m.p. 163–164 °C.

*(E)-5-(4-(Tributylstannyl)styryl)-1H-indole *(**11**). A mixture of **7** (29.8 mg, 0.1 mmol), bis(tributyltin) (290 mg, 0.5 mmol), and Pd(Ph_3_P)_4_ (12.0 mg, 0.01 mmol) in toluene (15 mL) was stirred at 110 °C overnight. After removing the solvent in vacuo, the crude products were purified by column chromatography (petroleum ether/AcOEt, 20/1) to give **11** as a light-yellow-colored solid with a yield of 22.3%. ^1^H-NMR (CDCl_3_) δ: 8.87 (1H, d, *J* = 2.8 Hz), 8.15 (1H, d, *J* = 8.1 Hz), 8.08 (1H, d, *J* = 8.7 Hz), 7.98 (1H, dd, *J_1_* = 8.9 Hz, *J_2_* = 1.6 Hz), 7.82 (1H, s), 7.52 (4H, dd, *J_1_* = 19.6 Hz, *J_2_* = 8.5 Hz), 7.40 (1H, dd, *J_1_* = 8.2 Hz, *J_2_* = 4.2 Hz),7.28 (2H, dd, *J_1_* = 16.4 Hz, *J_2_* = 8.0 Hz), 1.64–1.55 (6H, m), 1.40–1.35 (6H, m), 1.16–1.11 (6H, m), 0.97–0.90 (9H, m). ESI-MS *m/z* C_28_H_39_NSn found 510.4/calcd 509.2 ([M+H]^+^).

*Quinoline-6-carbaldehyde *(**13**). Quinoline-6-carbaldehyde (**13**) was prepared from 6-methylquinoline (**12**) according to the previously reported procedure [22]. 6-Methylquinoline (**12**, 4.0 g, 27.6 mmol) was heated to 160 °C and selenium dioxide (2.0 g, 18.4 mmol) was added. The mixture was stirred for 16 h, cooled to room temperature, and diluted with ethyl acetate (30 mL). The solution was decanted, and the residue was extracted with ethylacetate (20 mL × 2). The combined organic phase was concentrated, and the residue was purified by column chromatography on silica gel (petroleum ether/AcOEt, 4/1) to give **13** as a light gray solid (1.3 g, 30%). ^1^H-NMR (CDCl_3_) δ: 10.21 (1H, s), 9.06 (1H, dd, *J_1_* = 4.2 Hz, *J_2_* = 1.6 Hz), 8.38–8.35 (2H, m), 8.27–8.24 (2H, m), 7.55 (1H, dd, *J_1_* = 8.3 Hz, *J_2_* = 4.3 Hz).

#### 3.1.3. General procedure for preparing **14**–**19**

The suitable substituted compounds **2b**–**f** (1 mmol), **13** (1 mmol), CH_3_ONa (1 mmol) was heated to reflux in CH_3_OH (12 mL) for about 6 h. The organic solvent was removed under vacuum. Crude materials were washed by water and purified by column chromatography on silica gel (petroleum ether/AcOEt, 6/1).

*(E)-6-(4-Fluorostyryl)quinoline *(**14**). Yield 66.8%, ^1^H-NMR (DMSO-d_6_) δ: 8.86 (1H, dd, *J_1_* = 8.9 Hz, *J_2_* = 1.4 Hz), 8.35 (1H, d, *J* = 8.0 Hz), 8.11 (1H, dd, *J_1_* = 8.8 Hz, *J_2_* = 1.5 Hz), 8.07 (1H, s), 8.01 (1H, d, *J* = 8.8 Hz), 7.73(2H, dd, *J_1_* = 8.7 Hz, *J_2_* = 5.7 Hz), 7.54 (1H, dd, *J_1_* = 8.2 Hz, *J_2_* = 4.2 Hz), 7.45 (2H, dd, *J_1_* = 16.5 Hz, *J*_2_ = 7.7 Hz), 7.26 (1H, t, *J* = 8.8 Hz). HRMS *m/z* C_17_H_12_FN found 250.1039/calcd 250.1032 ([M+H]^+^). m.p. 120–121 °C.

*(E)-6-(4-Chlorostyryl)quinoline *(**15**). Yield 72.3%, ^1^H-NMR (DMSO-d_6_) δ: 8.86 (1H, d, *J* = 3.4 Hz), 8.35 (1H, d, *J* = 7.9 Hz), 8.12 (1H, d, *J* = 8.8 Hz), 8.08 (1H, s), 8.01 (1H, d, *J* = 8.7 Hz), 7.70 (2H, d, *J *= 8.2 Hz), 7.53 (2H, dd, *J_1_* = 8.4 Hz, *J_2_* = 4.1 Hz), 7.49–7.47 (4H, m). HRMS *m/z* C_17_H_12_ClN found 266.0732/calcd 266.0737 ([M+H]^+^). m.p. 129–130 °C.

*(E)-6-(4-Bromostyryl)quinoline *(**16**). Yield 73.9%, ^1^H-NMR (DMSO-d_6_) δ: 8.87 (1H, dd, *J_1_* = 4.1 Hz, *J_2_* = 1.5 Hz), 8.36 (1H, d, *J *= 8.0 Hz), 8.14 (1H, dd, *J_1_* = 8.8 Hz, *J_2_* = 1.6 Hz), 8.09 (1H, s), 8.01 (1H, d, *J* = 8.8 Hz), 7.63(4H, dd, *J_1_* = 13.3 Hz, *J_2_* = 8.8 Hz), 7.54 (1H, dd, *J_1_* = 8.1 Hz, *J_2_*= 4.2 Hz), 7.48 (2H, dd, *J_1_* = 16.6 Hz, *J_2_* = 13.3 Hz). HRMS *m/z* C_17_H_12_BrN found 310.0217/calcd 310.0231 ([M+H]^+^). m.p. 143–144 °C.

*(E)-6-(4-Iodostyryl)quinoline *(**17**). Yield 70.2%, ^1^H-NMR (DMSO-d_6_) δ: 8.87 (1H, d, *J* = 4.1 Hz), 8.35 (1H, d, *J* = 8.0 Hz), 8.12 (1H, d, *J *= 8.9 Hz), 8.09 (1H, s), 8.01 (1H, d, *J *= 8.9 Hz), 7.78 (2H, d, *J* = 8.0 Hz), 7.56–7.40 (5H, m). HRMS *m/z* C_17_H_12_IN found 358.0100/calcd 358.0093 ([M+H]^+^). m.p. 173–174 °C.

*(E)-6-(4-Methylstyryl)quinoline *(**18**). Yield 71.7%, ^1^H-NMR (DMSO-d_6_) δ: 8.85 (dd, *J_1_* = 4.0 Hz, *J_2_* = 1.4 Hz, 1H), 8.36 (d, *J *= 8.1 Hz, 1H), 8.11 (dd, *J_1_* = 8.8 Hz, *J_2_* = 1.6 Hz, 1H), 8.06 (s, 1H), 8.01 (d, *J* = 8.8 Hz, 1H), 7.57(d, *J *= 8.0 Hz, 2H), 7.54 (dd, *J_1_* = 8.3 Hz, *J_2_* = 4.3 Hz, 1H), 7.41 (s, 2H), 7.23 (d, *J* = 7.9 Hz, 2H). HRMS *m/z* C_17_H_12_IN found 246.1299/calcd 246.1283 ([M+H]^+^). m.p. 125–126 °C.

*(E)-6-(4-(Tributylstannyl)styryl)quinolin *(**19**). The same reaction described above to prepare **11** was used, and a primrose yellow-colored solid of **19** was obtained in a yield of 28.6% from **16**. ^1^H-NMR (CDCl_3_) δ: 8.87 (1H, d, *J* = 2.8 Hz), 8.15 (1H, d, *J* = 8.1 Hz), 8.08 (1H, d, *J* = 8.7 Hz), 7.98 (1H, dd, *J_1_* = 8.9 Hz, *J_2_* = 1.6 Hz), 7.82 (1H, s), 7.52 (4H, dd, *J_1_* = 19.6 Hz, *J_2_* = 8.5 Hz), 7.40 (1H, dd, *J_1_* = 8.2 Hz, *J_2_* = 4.2 Hz),7.28 (2H, dd, *J_1_* = 16.4 Hz, *J_2_* = 8.0 Hz), 1.64–1.55 (6H, m), 1.40–1.34 (6H, m), 1.16–1.13 (6H, m), 0.96–0.91 (9H, m). ESI-MS *m/z* C_29_H_39_NSn found 522.6/calcd 521.2 ([M+H]^+^).

#### 3.1.4. Preparation of Radioiodinated Ligands

The radioiodinated compounds [^125^I]**8** and [^125^I]**17** were prepared from the corresponding tributyltin derivatives by an iododestannylation according to the procedure described previously [21]. Brieﬂy, H_2_O_2_ (3%, 100 µL) was added to a mixture of a tributyltin derivative (0.1 mg/100 µL in ethanol), sodium [^125^I]iodide (speciﬁc activity 2,200 Ci/mmol), and 1 M HCl (100 µL) in a sealed vial. The reaction was allowed to proceed at room temperature for 15 min and then quenched by addition of saturated NaHSO_3_ solution (50 µL). The reaction mixture, after neutralization with 1 M NaOH, was puriﬁed by HPLC using a Alltech Alltima RPC-18 column (250 mm × 4.6 mm, 5 µm) and mobile phase consisting of acetonitrile with a flow rate of 1.0 mL/min. Finally, the radiochemical identity of the radioiodinated ligands were verified by co-injection and co-elution with non-radioactive **8** and **17** from HPLC profiles (Alltech Alltima RPC-18 column, 250 × 4.6 mm, 5 μm, CH_3_CN/H_2_O = 9/1 at the flow rate of 1.0 mL/min). The desired fractions containing the product were collectd and evaporated to dryness and redissolved in 100% ethanol. The ﬁnal products were stored at −20 °C for further studies.

### 3.2. Partition Coefﬁcient Determination

The determination of partition coefﬁcients of [^125^I]**8** and [^125^I]**17** was performed according to the procedure previously reported with some modifications [23]. Ligand [^125^I]**8** or [^125^I]**17** (~5 μCi**)** was mixed with 3 mL each of n-octanol and PBS (0.02 M, pH 7.4) in a test tube. The test tube was vortexed for 5 min at room temperature, followed by centrifugation for 10 min at 3,000 rpm. Two weighed samples from the *n*-octanol (50 µL) and buffer layers (400 µL) were counted in a γ-counter. The partition coefﬁcient was expressed as the logarithm of the ratio of the counts per gram from n-octanol *versus* that of PBS. Samples from the *n*-octanol layer were repartitioned until consistent partition coefﬁcient values were obtained. The measurements were done in triplicate and repeated three times.

### 3.3. *In Vitro* Binding Studies Using the Aggregated Aβ_1–40_

The lyophilized white powder of β-amyloid(1–40) were purchased from AnaSpec (San Jose, CA, USA). After reconstituted by adding basic buffer (1% NH_4_OH. 60–70 μL) to β-amyloid (1–40) (1 mg) aggregation of Aβ_1–40_ was carried out by gently dissolving Aβ_1–40_ (0.25 mg/mL) in a PBS buffer (pH 7.4). The solution was incubated at 37 °C for 72 h with gentle and constant shaking. Binding studies were carried out according to the procedure described previously with some modifications using [^125^I]TZDM as the radiolabeled standard [24]. Briefly, the competition binding assays were performed by mixing Aβ_1–40_ aggregates (100 µL), and [^125^I]TZDM (100 µL) in appropriate concentration (0.02 nM, diluted in 10% EtOH), test ligand (10^−5^–10^−10^ M, 100 µL) and PBS (0.02 M, pH 7.4, 700 µL) in a ﬁnal volume of 1 mL. The mixture was incubated at 37 °C for 2 h. Then the bound and free radioactivities were separated by vacuum ﬁltration through Whatman GF/B glass ﬁlters via a Brandel Mp-48T cell harvester followed by 3 × 4 mL washes with PBS (0.02 M, pH 7.4, 4 °C) containing 10% ethanol at room temperature. Filters containing the bound ^125^I ligand were counted in a γ-counter (WALLAC Wizard 1470, PerkinElmer Life Sciences, Waltham, MA, USA) with 75% counting efﬁciency. The IC_50_ values were determined using GraphPad Prism 5.0, and those for the inhibition constant (*K*_i_) were calculated using the Cheng Prusoff equation: *K*_i_ = IC_50_/(1 + [L]/*K*_d_) [25].

### 3.4. *In Vitro* Fluorescent Staining of Amyloid Plaques in Brain Sections from Transgenic Mouse

Parafﬁn-embedded brain sections of transgenic model mouse (8 µm, APP/PS1, 12 months, male) were used for the *in vitro *fluorescent staining of amyloid plaques. The brain sections were deparafﬁnized with xylene, ethanol and distilled water. After immersion in PBS (0.02 M, pH 7.4) for 30 min, the brain sections were incubated with 20% ethanol solution (1 µM) of compound **8** for 10 min. The localization of plaques was confirmed by staining with thioﬂavin-S on the adjacent sections. Finally, the sections were washed with 40% ethanol and PBS (0.02 M, pH 7.4). Fluorescent observation was performed by a Stereo Discovery V12 instrument (Zeiss, Oberkochen, Germany) equipped with a LP 505 filter set (excitation, 405 nm).

### 3.5. *In Vitro* Labeling of Brain Sections from Transgenic Mouse by Autoradiography

The brain sections mentioned above were incubated with [^125^I]**8** (5 µCi/100µL) for about 20 min at room temperature. Then the sections were washed with saturated Li_2_CO_3 _in 40% EtOH for 3 min and 40% EtOH for 3 min, followed by rinsing with water for 30 s. After drying, the ^125^I-labeled sections were exposed to phosphorus ﬁlm for 8 h and then scanned with the phosphor imaging system (Cyclone, Packard) at the resolution of 600 dpi. The presence and localization of plaques were confirmed by the ﬂuorescent staining with thioﬂavin-S on the same sections using a Stereo Discovery V12 (Zeiss) instrument equipped with a LP 505 filter set (excitation: 405 nm).

### 3.6. *In Vivo* Biodistribution in Normal Mice

*In vivo* biodistribution studies were performed in KunMing normal mice (female, average weight 18–22 g) and in accordance with the national laws related to the care and experiments on laboratory animal. A saline solution (100 µL) containing [^125^I]8 or [^125^I]17 (1 µCi) was injected directly into the tail vein of mice. The mice (n = 5 for each time point) were sacriﬁced at designated time points post-injection. The organs of interest were removed and weighed, and the radioactivity was counted with an automatic γ-counter (WALLAC Wizard 1470).

## 4. Conclusions

A series of (*E*)-5-styryl-1*H*-indole and (*E*)-6-styrylquinoline based compounds have been synthesized and evaluated as novel imaging probes for Aβ plaques. Compound **8** was found to possess nanomolar affinity for β-amyloid plaques. In autoradiography, [^125^I]**8** clearly labeled amyloid plaques in the cortex region of AD model mice. Moreover, [^125^I]**8** displayed high initial brain uptake and fast clearance in biodistribution studies in normal mice. The findings suggest that the (*E*)-5-styryl-1*H*-indole derivative [^123^I]**8** may be a potential probe for detecting β-amyloid plaques in the AD brain.

## Supplementary Materials

Supplementary materials can be accessed at: http://www.mdpi.com/1420-3049/17/4/4252/s1.
