# Supplementary material for: (E)-5-Styryl-1H-indole and (E)-6-Styrylquinoline Derivatives Serve as Probes for β-Amyloid Plaques
Source: Molecules. 2012 Apr 10;17(4):4252–65. doi: 10.3390/molecules17044252 (PMC6268995; doi:10.3390/molecules17044252)

Supplemental Material

Purity of (*E*)-5-Styryl-1*H*-indole and (*E*)-6-Styrylquinoline Derivatives together with HPLC Chromatograms

UV-wavelength: 254 nm
Alltech Alltima RPC-18 column (5 µm, ID = 4.6 mm, length = 250 mm)

Compounds	Flow rate (mL/min)	Mobile phase (CH_3CN/H_2O, v/v)	Retention time (min)	Purity (%)
**4**	1	70/30	9.793	100%
**5**	1	70/30	10.526	99.89%
**6**	1	70/30	15.957	99.05%
**7**	1	70/30	18.033	98.74%
**8**	1	70/30	20.316	96.39%
**9**	1	70/30	14.129	98.94%
**10**	1	70/30	10.214	98.51%
**14**	1	90/10	10.060	97.41%
**15**	1	90/10	15.706	99.81%
**16**	1	90/10	16.316	97.06%
**17**	1	90/10	19.550	95.71%
**18**	1	90/10	15.481	99.32%

Compound 4

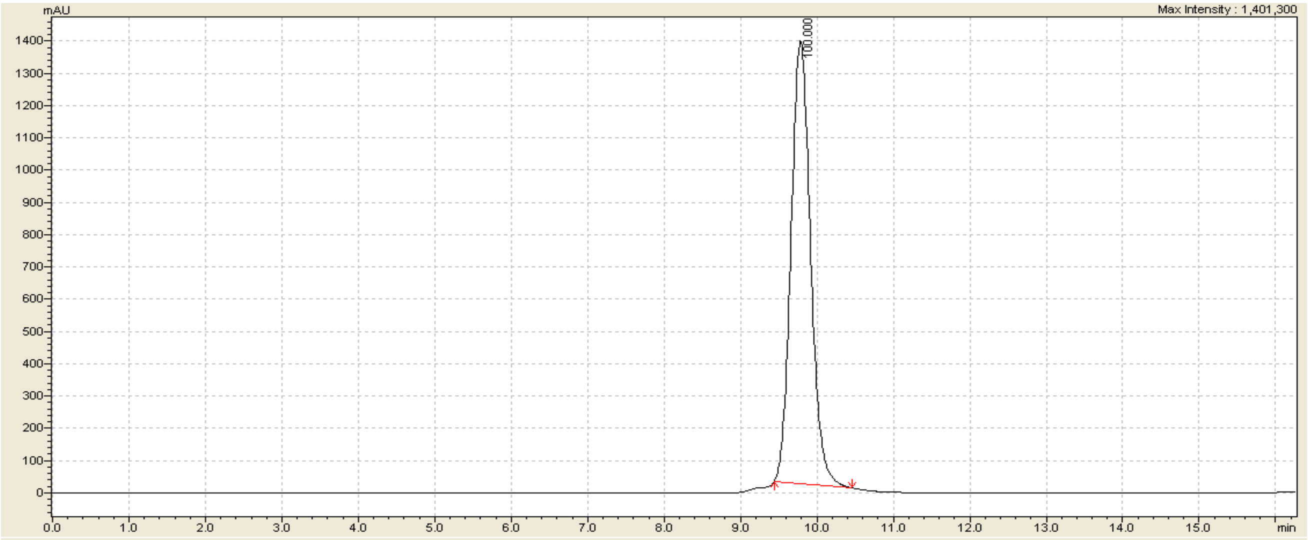

Compound 5

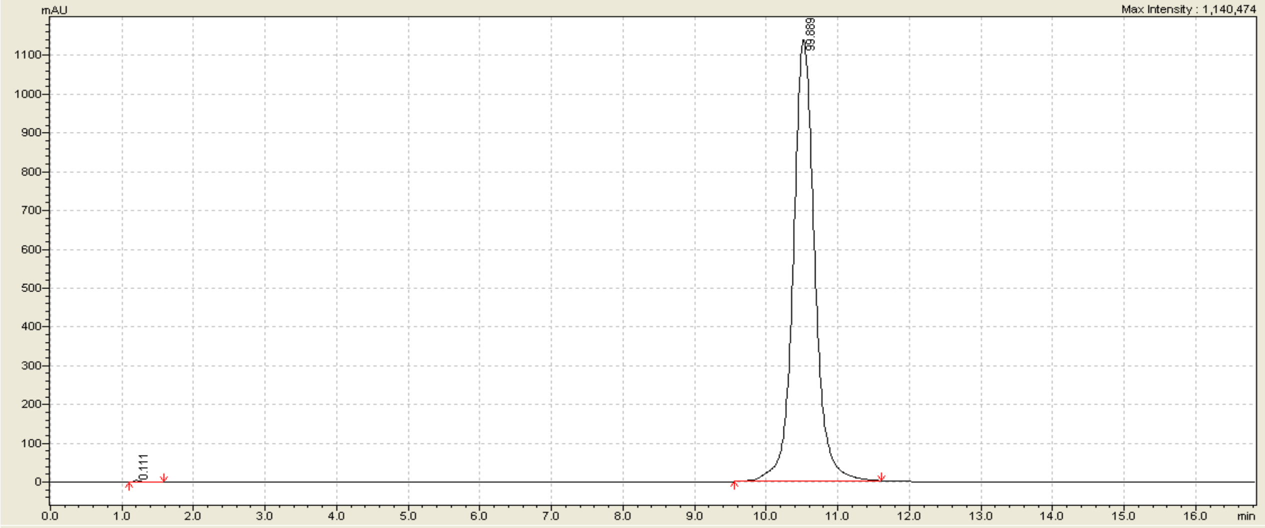

Compound 6

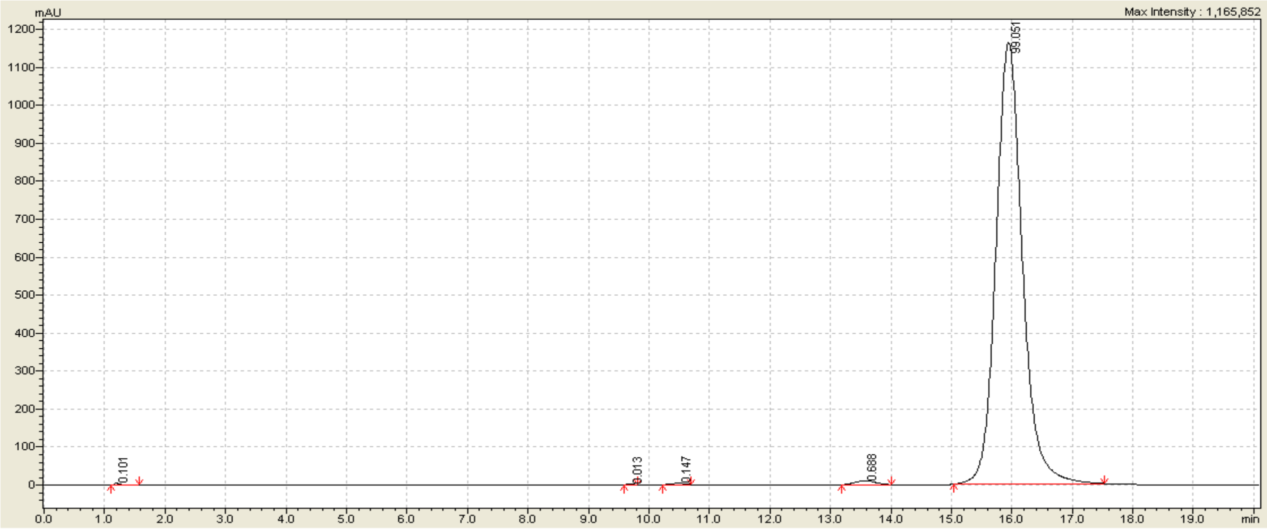

Compound 7

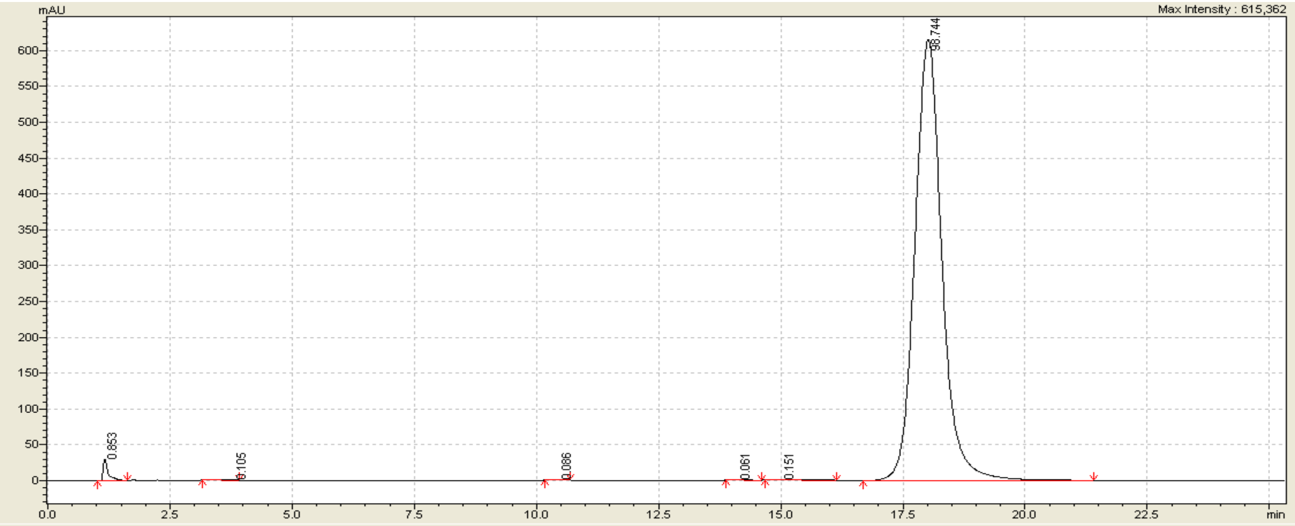

Compound 8

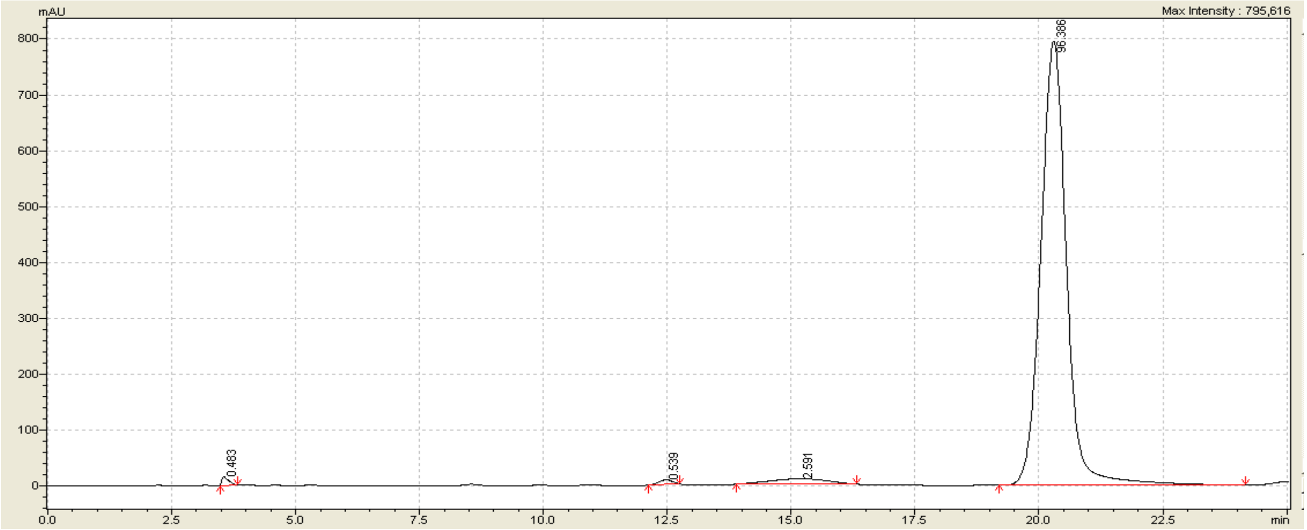

Compound 9

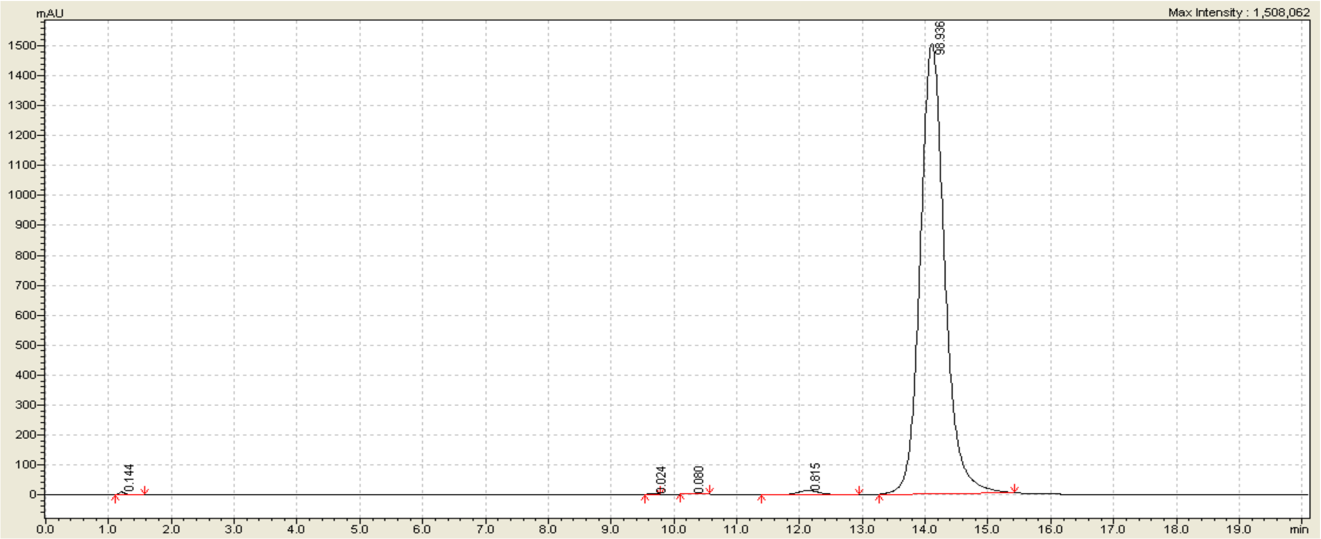

Compound 10

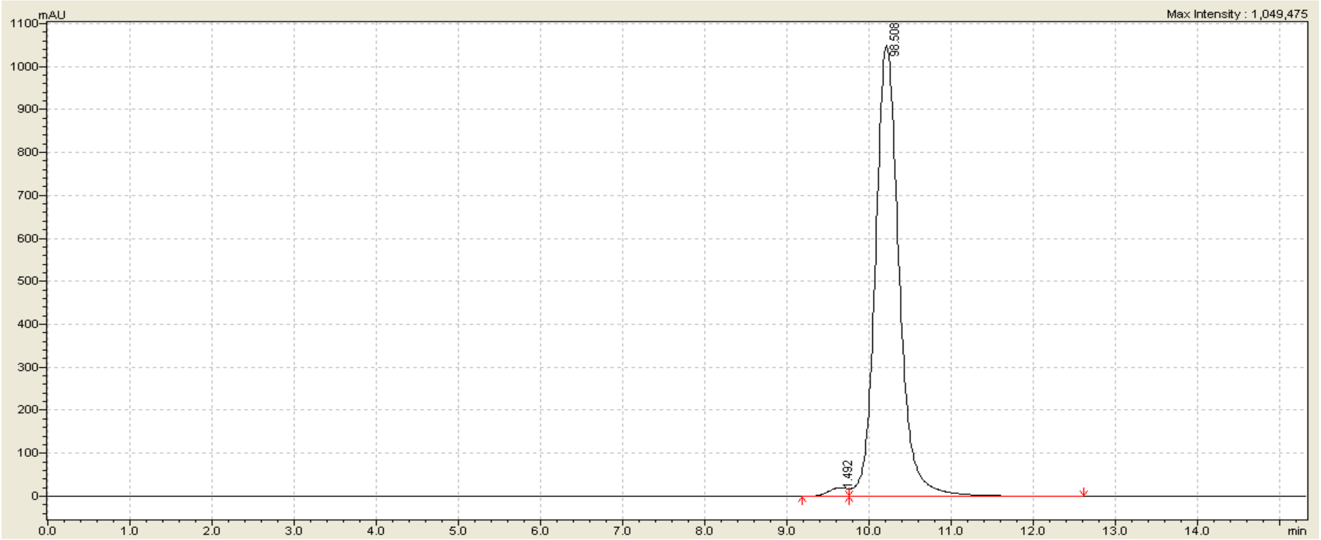

Compound 14

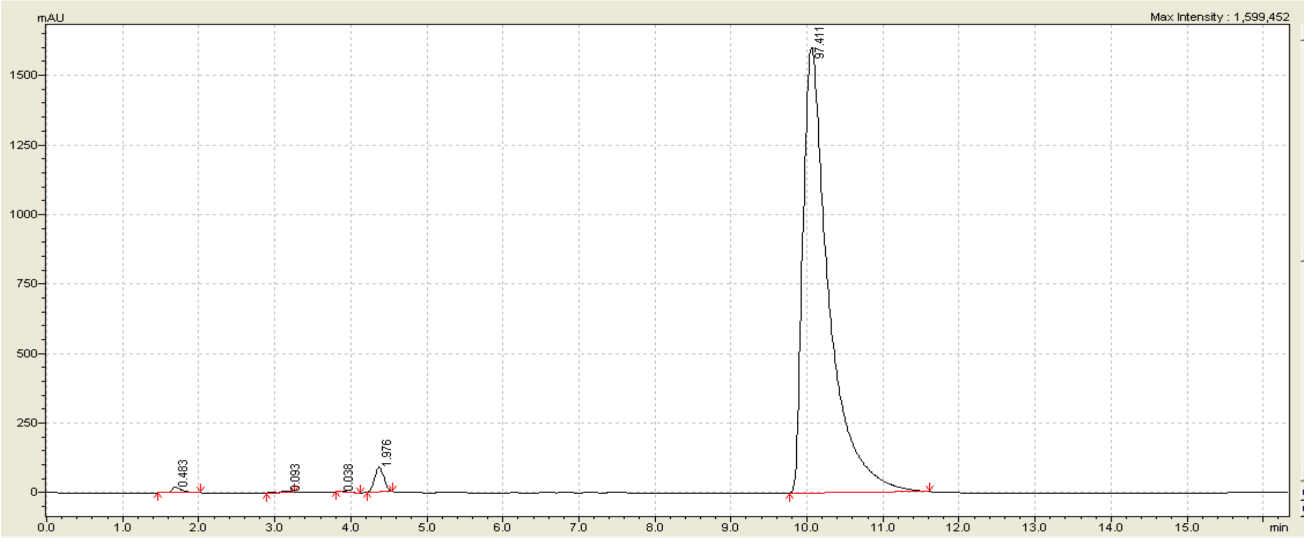

Compound 15

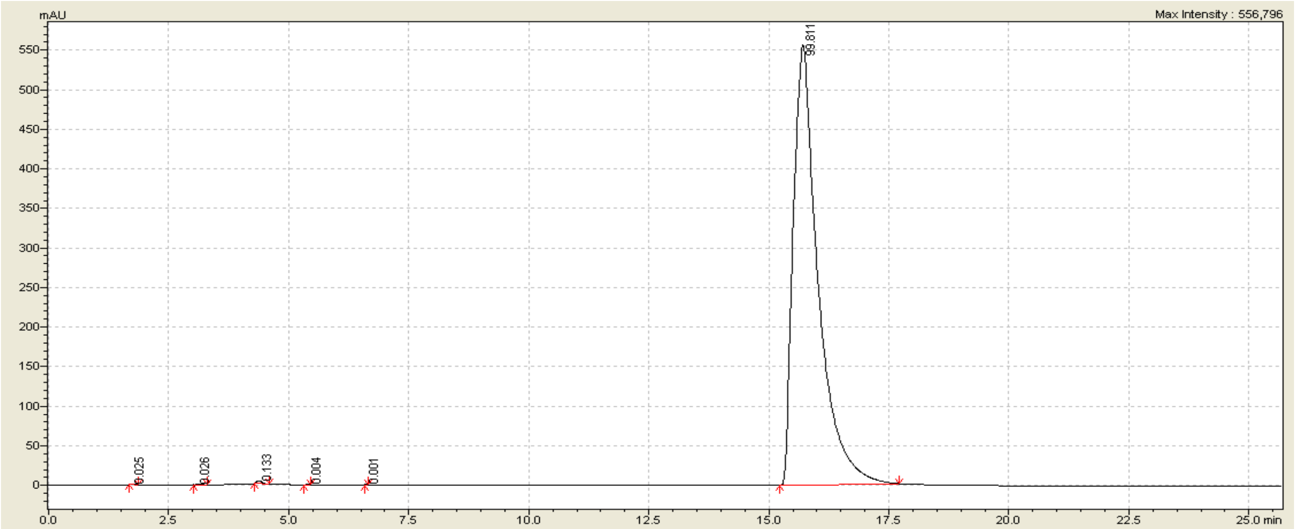

Compound 16

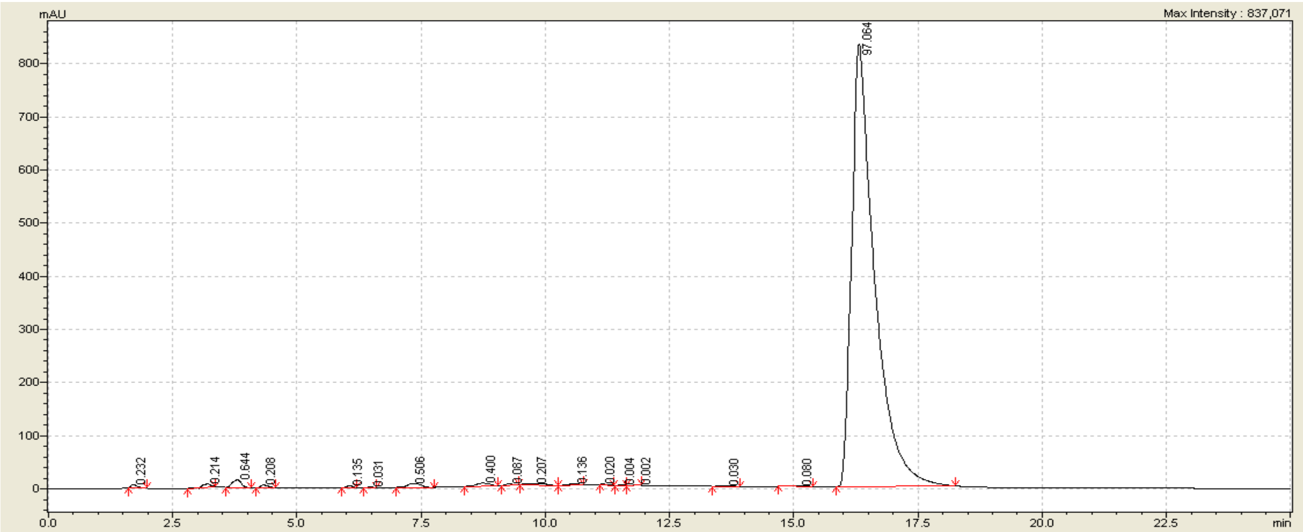

Compound 17

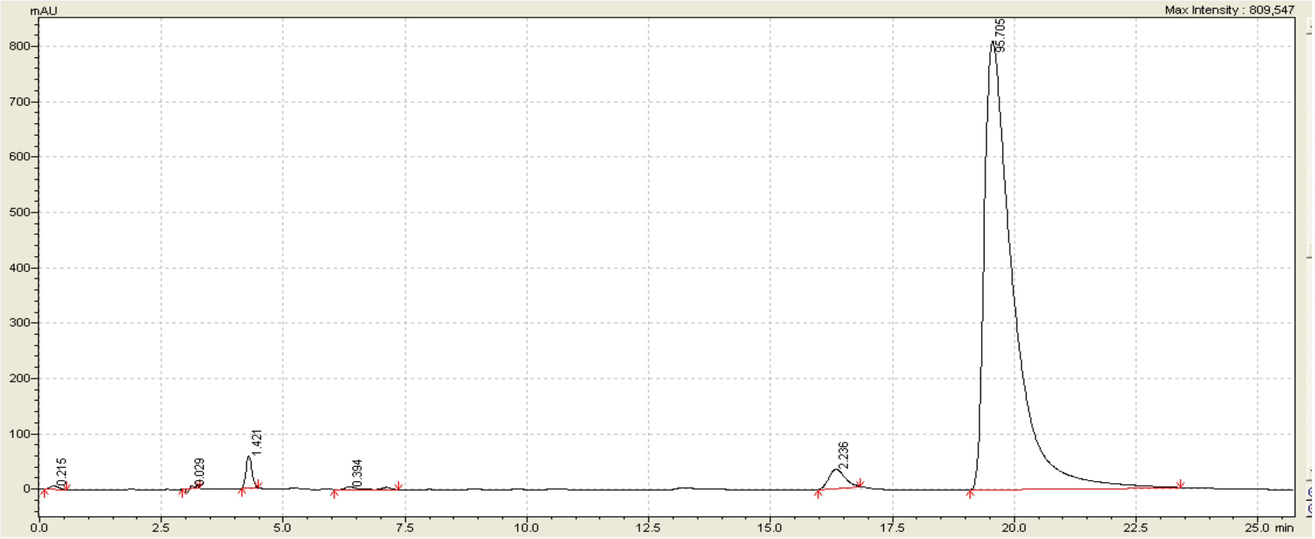

Compound 18

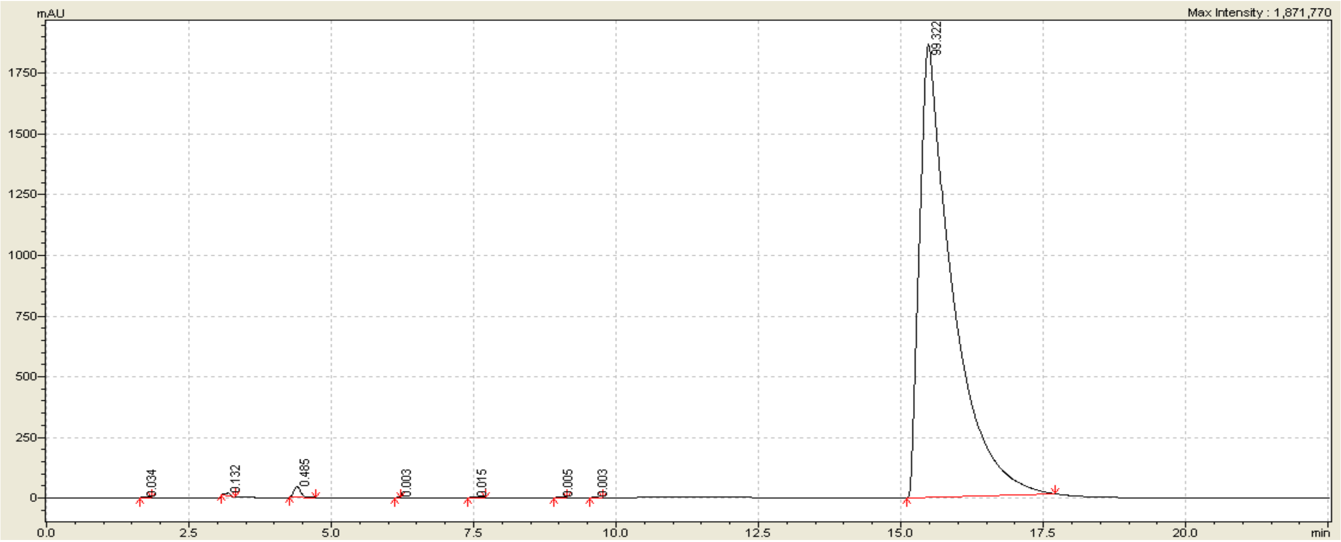

Supplement: Supplementary file 1 [file molecules-17-04252-s001.pdf]
